# Juvenile Psoriatic Arthritis: Myth or Reality? An Unending Debate

**DOI:** 10.3390/jcm12010367

**Published:** 2023-01-03

**Authors:** Roberta Naddei, Ana Rebollo-Giménez, Marco Burrone, Valentina Natoli, Silvia Rosina, Alessandro Consolaro, Angelo Ravelli

**Affiliations:** 1 Dipartimento di Scienze Mediche Traslazionali, Università degli Studi di Napoli Federico II, 80131 Naples, Italy; 2 Clinica Pediatrica e Reumatologia, IRCCS Istituto Giannina Gaslini, 16147 Genoa, Italy; 3 Dipartimento di Pediatria, Ospedale dei Bambini “Vittore Buzzi”, Università degli Studi di Milano, 20154 Milan, Italy; 4 Dipartimento di Neuroscienze, Riabilitazione, Oftalmologia, Genetica e Scienze Materno-Infantili (DiNOGMI), Università degli Studi di Genova, 16132 Genoa, Italy; 5 Direzione Scientifica, IRCCS Istituto Giannina Gaslini, 16147 Genoa, Italy

**Keywords:** juvenile idiopathic arthritis, juvenile psoriatic arthritis, psoriasis, childhood arthritis, classification

## Abstract

Juvenile psoriatic arthritis (JPsA) accounts for 1–7% of all cases of juvenile idiopathic arthritis (JIA) and its definition has been a matter of controversy among pediatric rheumatologists for many years. The traditional attribution of JPsA to the spondyloarthropathy group was challenged in the early 1990s, whereas the recent demonstrations of its heterogenous nature have led to questions about its identification as a distinct category in JIA classification. It has been shown that children with the phenotype of JPsA can be divided in two subgroups, one presenting with the features of early-onset ANA-positive JIA, and another that belongs to the spectrum of spondyloarthropathies. The few studies that have compared the clinical characteristics and genetic determinants of JPsA with those of the other JIA categories have obtained contrasting findings. The debate on the categorization of JPsA as a distinct entity within JIA classification is still ongoing and has prompted the revision of its current classification.

## 1. Introduction

Juvenile idiopathic arthritis (JIA) is an umbrella term that encompasses all forms of arthritis of unknown origin that last for >6 weeks and have their onset before 16 years of age. It is the most common chronic inflammatory rheumatic condition in childhood and is a leading cause of acquired disability in the pediatric period [1,2,3]. Juvenile psoriatic arthritis (JPsA) is one of the seven JIA categories outlined by the International League of Associations for Rheumatology (ILAR) classification for JIA [4], and accounts for 1–7% of all cases of JIA (Table 1) [5].

The concept of JPsA has been a matter of debate for many years among pediatric rheumatologists [6,7,8,9,10]. The initial area of controversy regarded the attribution of JPsA to the spectrum of spondyloarthropathies and its relationship to the forms of adult psoriatic arthritis that belong to this disease family. More recently, the characterization of JPsA as a single homogeneous disease entity has been challenged as many studies have shown considerable heterogeneity within its clinical phenotype. These controversies are reflected in the evolution of the classification of JPsA over the years. In a recent Delphi survey conducted by the Pediatric Rheumatology International Trials Organization (PRINTO) in the context of an effort aimed to revise the current classification criteria for JIA, no consensus was reached on the definition of JPsA [11]. In this review, we re-examine the history of the concept of JPsA, describe the proposed definitions and classifications, and summarize the main findings of the studies that have addressed these uncertainties.

## 2. Methods

For this narrative review, we conducted a comprehensive PubMed search of full-length articles in English using the following key terms: ‘juvenile arthritis and psoriasis’ or ‘juvenile psoriatic arthritis’. We excluded articles published in non-peer reviewed journals. Papers were independently reviewed by at least two authors, and those judged more informative for the purpose of the review were retained. The list of references of the relevant articles was also manually searched to find other potentially suitable studies. Review articles and book chapters are also cited to provide readers with more details.

## 3. Review

### 3.1. History of JPsA Classification

In 1962, Ansell and Bywaters published the first observation of psoriasis in seven children with chronic arthritis [12]. Some years later, Angevine and colleagues described the case of a young boy with psoriasis who developed a persistent non-migratory polyarthritis, and highlighted the similarity between this patient’s disease and adult psoriatic arthritis [13]. JPsA was initially grouped within the seronegative spondyloarthropathies, like its adult counterpart [14]. As a result, it was not included in the American College of Rheumatology (ACR) classification for juvenile rheumatoid arthritis, which excluded the spondyloarthropathies [15], whereas it was placed within the spondyloarthropathies category in the European League for Rheumatology juvenile chronic arthritis classification [16].

In 1976, the first case series of JPsA was reported by Lambert and colleagues, who retrospectively evaluated 43 children [17]. An important observation from that study was that arthritis preceded psoriasis in approximately half the patients. The authors proposed the first definition of psoriatic arthritis in childhood, by stating that JPsA could be diagnosed in any child with arthritis who develop psoriasis at any time up to 15 years after the onset of joint disease. This proposal was supported by a subsequent case series of 60 patients with JPsA by Shore and Ansell [18], who observed that a family history of psoriasis, the presence of nail pits, and a cumulative asymmetrical arthropathy could be predictive of JPsA before psoriasis onset. However, it was noticed that the application of the definition by Lambert et al. only seldom allowed the diagnosis of JPsA early in the disease course [18,19].

To address the concern that JPsA could be underdiagnosed in case of delayed occurrence of the typical psoriatic rash, Southwood and co-workers devised a new definition of JPsA, based on the so called “Vancouver criteria” (Table 2), which was aimed to enable the diagnosis of JPsA in the absence of overt psoriasis [19]. These criteria allowed the definition of JPsA in the presence of arthritis and a psoriasis-like rash or other clinical features, including dactylitis, nail pits, or a family history of psoriasis. The new categorization led to the identification of a population of patients of a younger age compared to those reported in previous series [20].

Southwood et al. also noticed that JPsA was more similar to classic JIA than to spondylarthritis and, thus, questioned the inclusion of JPsA within the spectrum of these conditions [19]. This view was reinforced by Ross Petty who, in a review published in 1994 [6], criticized the inclusion of JPsA within the spondyloarthritis group based on the following considerations: (i) the higher frequency of female sex and antinuclear antibody (ANA) positivity in JPsA compared to spondyloarthropathies; (ii) the lower prevalence of positive HLA-B27, axial skeleton arthritis, and enthesitis, which are hallmarks of spondyloarthritis, in JPsA; (iii) the different characteristics of uveitis, which is an important complication of both conditions, but is typically acute and self-limited in spondyloarthritis and chronic in JPsA. However, the pattern of joint disease, especially the presence of dactylitis and the asymmetry of arthritis affecting both large and small joints, led Petty to also set JPsA apart from oligoarticular JIA [6].

**Figure 1:**
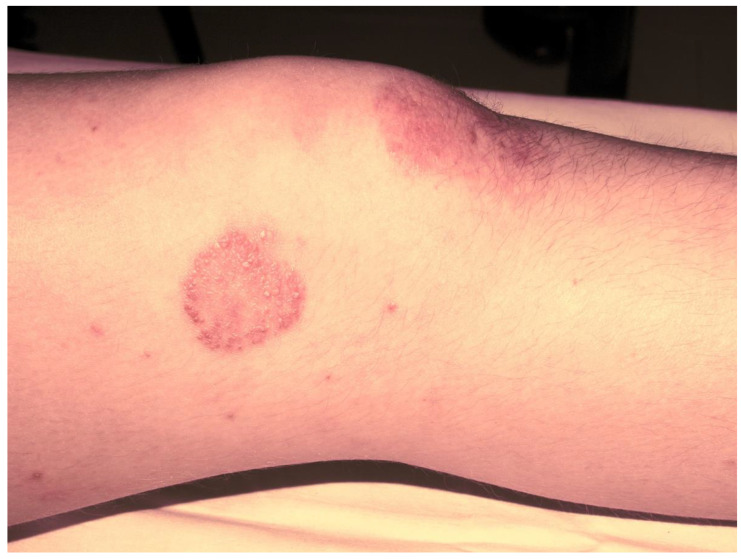
Psoriatic skin lesion in a child with JPsA.

**Figure 2:**
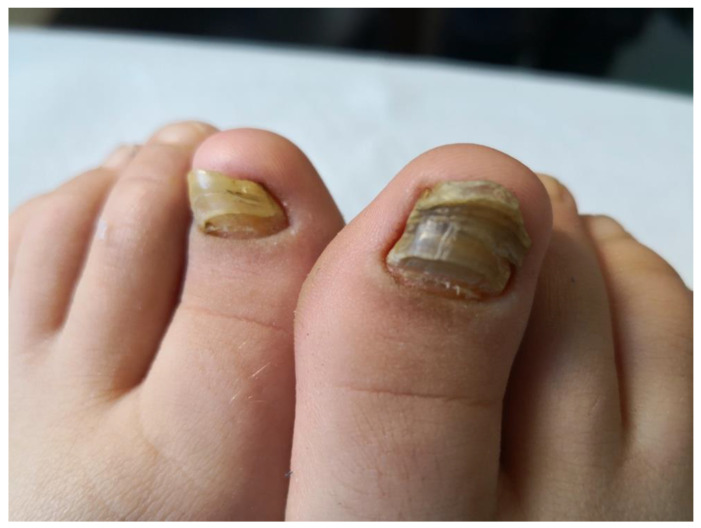
Nail changes in a child with JPsA.

Subsequently, the ILAR Pediatric Task Force outlined the JPsA category in the JIA classification, which was promulgated in 1994 [21], and revised in 1997 [22] and then in 2001 [4] (Table 2). The diagnosis of JPsA by ILAR criteria requires the simultaneous presence of arthritis and a typical psoriatic rash (Figure 1) or, if the rash is absent, the presence of arthritis and any two of the following: family history of psoriasis in a first-degree relative; dactylitis (swelling of one or more fingers that extends beyond the joint margins); and nail pitting (pits in at least two nails) (Figure 2). With the aim of delineating homogeneous and mutually exclusive categories of JIA, the ILAR criteria did not allow the diagnosis of JPsA in patients with positive rheumatoid factor (RF) test, family history of an HLA-B27-associated disorder in a first-degree relative, onset of arthritis in a HLA-B27 positive male aged more than 6 years, or systemic arthritis [4].

Recently, the research network PRINTO challenged the validity of the ILAR classification system, especially its assumption of defining homogeneous disease categories, and proposed new preliminary criteria for JIA aimed to identify more homogeneous entities [11]. These criteria were developed through international expert consensus and will be formally validated by means of a prospective collection of clinical and laboratory data, which is currently ongoing. Notably, no consensus was reached on a provisional definition of JPsA, and the criteria for this condition were intended to be established after the analysis of prospective data.

### 3.2. The Clinical Spectrum of JPsA

The clinical features of JPsA appear to be quite heterogeneous [23]. The age at onset is bimodally distributed, with a first peak occurring during the preschool years and a second during mid-adolescence [19,24,25]. In about 80% of children with JPsA, joint disease begins as an oligoarthritis, and the onset is not uncommonly marked by a monoarthritis [23]. Even though the most affected joint is the knee, followed by the ankle, most patients develop arthritis of both small and large joints, with an asymmetrical distribution [19,25,26,27]. Without an effective treatment, progression from oligoarticular to polyarticular involvement is observed in 60–80% of patients [18,19,20]. Axial disease is seen in up to 25% of patients [18,25], mainly with asymmetric sacroiliitis. Dactylitis is recorded in 20–40% of children with JPsA; it usually affects one or a few digits, and can be symptomatic or asymptomatic [23]. Dactylitis consists of the variable combination of inflammatory synovial and extra-synovial digital lesions, such as flexor tenosynovitis, enthesitis, and peritendinous tissue inflammation [28,29]. In contrast with the adult population with psoriatic arthritis, bone erosions are not commonly observed in the hand joints of children with JPsA [28].

Overt psoriasis is seen in 40–60% of patients with JPsA [23], and occurs after the onset of arthritis in about half of the cases [27]. Nail changes, including pits, onycholysis, horizontal ridging and discoloration, are detected in 50 to 80% of patients (Figure 2) [23]. Chronic uveitis occurs in 10–15% of children with JPsA, and is indistinguishable from that seen in oligoarticular and polyarticular arthritis; acute anterior uveitis is rare [19,20,26,30]. Laboratory findings are generally unremarkable, with the ANA test being positive in about 50% of cases [19,20,24].

Based on the review of the existing literature, Martini noticed in an editorial published in 2003 [7] that early-onset JPsA is more common in young girls presenting with an asymmetric oligoarthritis that can extend over time; in these patients, chronic anterior uveitis occurs with a frequency similar to that seen in early-onset oligoarticular JIA (roughly 20%) and is associated with ANA positivity [31]. Conversely, the older group of children with JPsA has a male predominance and present some features typical of enthesitis-related arthritis (ERA) (the pediatric counterpart of adult undifferentiated spondyloarthritis), such as axial involvement and enthesitis. These observations were later corroborated by Stoll and colleagues in a retrospective study that described two distinct populations of patients within a cohort of 139 patients meeting the Vancouver criteria for JPsA [24]. Children with early disease onset were more likely to be female, to have dactylitis and polyarticular onset, and to be ANA positive, but less likely to have frank psoriasis, enthesitis, or axial disease, which were mainly detected in patients with late-onset JPsA. Similar findings were observed when the data were reanalyzed using the stricter ILAR criteria [32]. The recent analysis of 361 children of the JPsA cohort recruited within the Childhood Arthritis and Rheumatology Research Alliance (CARRA) Registry also supports the dissection of patients with JPsA into two clinical subgroups, each characterized by a different age at onset and the respective above-mentioned clinical features [25].

A number of studies have shown a wide, though still unexplained, variability of the frequency of the JIA categories, including JPsA, across geographic areas and ethnic groups [33]. The epidemiology of the JIA categories was recently investigated in a more systematic way in the EPOCA (EPidemiology, treatment, and Outcome of Childhood Arthritis) study [5], a multinational, cross-sectional, observational cohort investigations that collected the demographic, clinical, and therapeutic data of 9081 patients with JIA living in 49 countries. The frequency of JPsA among the total cases of JIA ranged from 1.3% in Southeast Asia to 7.1% in North America (Table 3). The frequency of uveitis in JPsA was also variable, with figures similar to those of oligoarthritis, RF-negative polyarthritis, and undifferentiated arthritis in southern and western Europe and lower than those of these categories in other geographical areas. Altogether, these findings suggest that some clinical features of children currently classified as JPsA may differ throughout the world (Table 3).

### 3.3. Genetics of JPsA

Data from genetic analyses conducted specifically in JPsA patients are available. Some single-nucleotide polymorphisms (SNPs) have been found to be associated with JPsA. Using the same approach of testing genetic associations previously applied in adult psoriatic arthritis in 1244 JIA cases, of whom 93 had JPsA, and 5200 controls, Hinks et al. identified a negative association between JPsA and a SNP linked to the minor protective allele of interleukin 23R, the IL-23 receptor gene, which was not observed in the overall JIA cohort or in different JIA subtypes [34]. Furthermore, two SNPs, rs224204 in the MEFV gene and rs3806265 in the NLRP3 gene, were found to be associated with JPsA by Day et al., who tested genes that cause monogenic disorders with some phenotypic overlap with JIA [35]. However, the majority of genetic data on JPsA refers to the study of HLA associations and their results are not conclusive. The first of such investigations, published in 1993 by Ansell et al., describes a statistically significant association with HLA-B27 in 70 patients with JpsA compared to 310 controls [31]. Several years later, Flatø and coworkers showed that the HLA-DRB1*11/12 status differentiates JPsA patients from those with either oligoarthritis or polyarthritis [36]. In a more recent study by Hinks and colleagues describing the HLA associations across each JIA category in 5043 JIA cases, 112 of whom had JPsA, diverse associations with HLA alleles and JPsA were found: HLA-DQA1*0401 (*p* = 0.0001), HLA-DRB1*08 (*p* = 0.0003), HLA-DQB1*0402 (*p* = 0.0008), HLA-C*0602 (*p* = 0.008), and HLA-B*27 (*p* = 0.003). However, none of those associations reached the genome-wide level of significance (*p* < 5 × 10^−8^) [37]. According to authors’ interpretation, the observed mixed HLA associations in JPsA could be explained as the result of some misclassification (that is, the JPsA sample might have contained subjects with oligoarthritis, RF-negative polyarthritis, or ERA categories). It could, however, be argued that these findings reflect the heterogeneity of the JPsA category, as defined by ILAR criteria.

### 3.4. Treatment of JPsA

Only a few data are available on the specific management of JPsA and the overall treatment approaches for this illness do not differ from those used in other JIA subtypes. Notably, the recently updated ACR guidelines for the treatment of non-systemic JIA were based on broader clinical phenotypes, rather than on ILAR categories, and were guided by the number of involved joints, the presence of sacroiliitis or enthesitis, and the involvement of the temporomandibular joint [38,39].

The following therapeutic choices recommended by these guidelines can be applied to JPsA clinical phenotypes. Intra-articular glucocorticoids and nonsteroidal anti-inflammatory drugs (NSAIDs) are recommended as first-line therapy in oligoarthritis, and as adjunct therapy in polyarthritis. Conventional synthetic disease-modifying antirheumatic drugs (csDMARDs), with methotrexate as the preferred agent, should be started in the case of inadequate response to the first-line treatment in oligoarthritis and as an initial therapy in polyarthritis. Treatment with a biological DMARD (bDMARD) is recommended in case of incomplete response to (or intolerance of) csDMARDs, in patients with active sacroiliitis and enthesitis despite NSAIDs or, as part of initial therapy, in the presence of high disease activity and risk factors in polyarthritis [38,39].

Data on the use of the tumor necrosis factor inhibitor (TNFi) etanercept (ETN) in JPsA have been provided by the CLIPPER study, which demonstrated the effectiveness and safety of ETN in a JIA cohort including patients with extended oligoarticular JIA, ERA, and JPsA [40,41,42]. Among other TNFi, adalimumab and infliximab are the preferred agents when active uveitis is present, irrespective of the ILAR category, in the case of incomplete response to topical therapy and csDMARDs or in the presence of sight-threatening complications [43]. Although TNFi are the most frequently used bDMARDs in children, non-TNFi agents of proven efficacy in the treatment of JIA, such as abatacept or tocilizumab, may be used or preferred in certain scenarios, based on patient features and preferences [38,39].

In the last few years, the introduction of small molecules, such as Janus kinase (JAK) inhibitors, hold promise for the treatment of chronic arthritis. A pivotal trial has recently shown the effectiveness of tofacitinib, an oral JAK inhibitor, in a cohort of patients with polyarticular course JIA, including 20 subjects with JPsA [44]. This molecule has been recently approved by European Medicine Agency (EMA) for the treatment of polyarticular JIA and JPsA. The blockade of the IL-17 and IL-12/23 pathways has shown robust therapeutic effect in adult psoriasis and PsA. A monoclonal antibody against IL17A, secukinumab, is currently approved by the EMA for refractory PsA and ankylosing spondylitis in adults, and has yielded significantly longer time to disease flare than placebo and a good safety profile in children with ERA and JPsA in a randomized phase 3 trial [45]. A multicenter, open-label trial (ClinicalTrials.gov Identifier: NCT04527380) is currently ongoing to assess the efficacy and safety in children with ERA and JPsA of another anti-IL17A, ixekizumab, which has already been approved for the treatment of refractory PsA and axial spondyloarthritis, and adult and juvenile plaque psoriasis. Recently, the US Food and Drug Administration approved an anti-IL-12/23 monoclonal antibody, ustekinumab, for the treatment of patients with JPsA 6 years or older, based on the extrapolation [46] of data from studies about the use of ustekinumab in pediatric patients with psoriasis, including a small sample of JPsA [47,48] and in adults with PsA [49].

### 3.5. JPsA vs. Adult-Onset PsA

While the prevalence of arthritis in children with psoriasis is approximately 2% [50], up to 30% of adult patients with psoriasis develop joint inflammation. In PsA, unlike JPsA, the onset of psoriasis generally precedes that of arthritis, which usually occurs within 10 years after the diagnosis of psoriasis [51,52]. Peripheral arthritis is the most common presentation both in adults and children, whereas axial disease is more frequent in adults. Dactylitis is a distinctive feature of PsA, both in pediatric and adult populations, while solely the late-onset subgroup of JPsA displays enthesitis. Adult PsA often pursues a severe course, with a high frequency of bone and cartilage destruction in the joints and pathologic new bone formation [53]. A sizeable proportion of patients (about 25%) with JPsA develop radiological articular damage [19,25], although bone erosions are not commonly observed in the hand joints of children with JPsA [28].

The ClASsification criteria for Psoriatic ARthritis (CASPAR), which were developed to standardize enrollment in PsA clinical trials, provide guidance for PsA diagnosis in adulthood [54]. These criteria incorporate cutaneous and radiographic features and musculoskeletal manifestations, including both peripheral and axial arthritis [54]. In contrast, according to ILAR criteria, many children with axial arthritis are classified as having ERA or undifferentiated JIA [4]. The application of CASPAR criteria to the CARRA JIA cohort led to the identification of additional patients who would be classified as PsA by adult rheumatologists; those patients were likely to have less psoriasis but more enthesitis and sacroiliitis/inflammatory back pain, which are manifestations of spondyloarthritis [25]. Notably, in a study aimed to determine how JIA patients older than 18 fulfil the classification criteria for adult rheumatic diseases, all patients with JPsA fulfilled the CASPAR criteria for PsA [55].

RF is typically negative in both conditions and its presence excludes the diagnosis of JPsA by ILAR criteria and its absence is one of the CASPAR criteria for PsA [4]. RF positivity is reported in about 5% of cases in both PsA and JPsA. In line with what is observed in JPsA, the prevalence of ANA in PsA has been reported to be about 50% [56].

## 4. The Ongoing Debate on JPsA: Juvenile Psoriatic Arthritis or Juvenile Arthritis with Psoriasis?

The clinical heterogeneity of JPsA had shed doubt on the appropriateness of its inclusion as a single category within the spectrum of JIA. The first to address this issue was Petty in the aforementioned review published in 1994 [6], where he explained the nature of the relationship between psoriasis and arthritis in childhood in two different ways. Some patients diagnosed as having JPsA may have the coincidental association of psoriasis and JIA or spondyloarthritis. The other patient group has a distinctive arthropathy characterized by asymmetric oligoarticular disease involving large and small joints, with or without dactylitis, which could represent “true” JPsA and should be classified separately from both the other JIA subgroups and spondyloarthritis. The few studies that have compared the clinical characteristics and genetic determinants of JPsA with those of the other JIA categories have obtained contrasting findings. Therefore, whether JPsA should be placed as a distinct category in JIA classification is still debated. Some authors question that children with arthritis and psoriasis (or a psoriatic “diathesis”) constitute a single homogeneous population, and that JPsA should be treated as a single disease entity [7,9,57], as it is done in the current ILAR classification [4]. On the other hand, based upon studies reporting clinical differences between JPsA and non-psoriatic arthritis and some distinctive genetic markers, other authors argue that JPsA presents sufficient unique characteristics to be placed among the spectrum of JIA as a distinctive category [8,10].

### 4.1. JPsA Is a Distinct Entity within the JIA Spectrum

By comparing children with oligoarticular JIA and JPsA, Stoll et al. [58] found that children with JPsA were older at onset and more likely to present with small joints and wrists involvement, as previously reported by Huemer and colleagues [26], and to experience a polyarticular course of arthritis, as observed by Ekelund et al. [59]. The same clinical differences were found when the comparison was restricted to children with an onset age under 5 years [58]. Although children with late-onset JPsA were found to share some ERA features, such as enthesitis and axial involvement, the same patients had a relatively lower frequency of HLA-B27 positivity, sacroiliitis, and hip arthritis as compared to ERA. This observation has led some authors to consider children with late-onset JPsA distinct from their counterparts with ERA [10,25], a view shared by Zisman and colleagues in the report by the Group for Research and Assessment of Psoriasis and Psoriatic Arthritis (GRAPPA) in its 2017 annual meeting [60]. It should be considered, however, that the low frequency of spondyloarthritis features in JPsA could be inherent in its definition, because these features are inclusion criteria for ERA and exclusions for JPsA in the ILAR classification.

With regard to JPsA outcome, an analysis of 336 JIA patients, 31 of whom had JPsA, evaluated by Flatø and colleagues after a median disease duration of 23 years, showed that JPsA was associated with an unfavorable outcome compared to the other JIA categories in terms of physical health self-assessment and need for disease-modifying anti-rheumatic drugs and/or anti-tumor necrosis factor agents [36]. Similar results have been reported by Ekelund and colleagues, who found that JIA patients with psoriatic features had a higher number of affected joints and a higher rate of active disease at an 8-year follow-up compared to the rest of the cohort in a Nordic JIA population [59]. In other studies, children with JPsA had a higher risk of overweight and obesity compared to oligo- or polyarthritis patients [61,62], similar to what was described in the adult population with psoriatic arthritis [63]. Moreover, the observation that JPsA shares some clinical features with its adult counterpart and that most children with JPsA will eventually carry the diagnosis of PsA in adulthood [55] may support the usefulness of maintaining JPsA as a single disease entity within JIA, as claimed by Stoll and Mellins [10]. A collaborative effort between adult and pediatric rheumatologists has been called for by the GRAPPA group to raise awareness of the differences in JPsA and PsA classification, to validate the CASPAR criteria across the age spectrum, and to ensure a successful transition of young adults with JPsA to adult care [60].

From a genetic point of view, as mentioned above, specific associations between JPsA and SNPs in the IL-23 receptor gene, MEVF, and NRLP3 and diverse associations with HLA alleles have been found, even though none of HLA associations has reached the genome-wide level of significance in the study by Hinks et al. [34,35,36,37].

### 4.2. JPsA Is JIA with Psoriasis

In 2003, Martini put forward his view of JPsA as a heterogeneous condition [7]. His interpretation was that JPsA is not a unique condition, but includes two different entities. The first is predominant in females and is characterized by early onset, asymmetric arthritis, ANA positivity, and a high risk of iridocyclitis. It closely resembles early-onset oligoarticular JIA, in which the concurrent psoriasis slightly modifies the disease expression, with higher frequency of dactylitis and small joint involvement, and a more rapid spread of arthritis. The other entity shares the features of ERA, is part of the spectrum of spondyloarthritis, and is similar to the forms of adult psoriatic arthritis that belong to the same disease family. Martini concluded that the “cluster of features” that consists of asymmetric arthritis, early onset, female predominance, ANA positivity, high risk of chronic anterior uveitis, and association with HLA-DR8, may be a more meaningful tool to define a homogenous group of patients, rather than the presence of psoriasis. Martini also criticized that the ILAR criteria lead patients with the simultaneous presence of JPsA and ERA to fall into the “undifferentiated arthritis” category, thus excluding children with spondyloarthropathic features from the JPsA group [32,57].

Compelling evidence of the similarity between early-onset JPsA and early-onset non-psoriatic JIA was provided by a study published by Ravelli et al., which substantiated the hypothesis of the “cluster of features”. A total of 860 children with JIA (oligoarticular, rheumatoid factor-negative polyarticular, psoriatic, and undifferentiated) were included in a retrospective analysis and divided in two groups based on their ANA status, using a cut-off titer of 1:160 [64]. The whole group of ANA positive patients, evaluated together irrespective of the ILAR category, shared similar clinical features, including strong female predominance, early onset of disease, asymmetric arthritis, and high risk of chronic iridocyclitis. This finding indicated that ANA-positive patients with JIA constitute a homogeneous subgroup, irrespective of the course of joint disease and the presence of psoriasis.

This assumption was corroborated by a recent cross-sectional analysis of 1862 German patients with JPsA, aimed to investigate the risk factors for uveitis in this JIA subset. Ocular involvement was found to be more frequent in females (73.0 vs. 62.9%, *p* = 0.03), in ANA-positive patients (60.3 vs. 37.0%, *p* < 0.001), and in patients with younger age at disease onset (5.3 ± 4.1 vs. 9.3 ± 4.4 years, *p* < 0.001), demonstrating that the main features of children with JPsA who develop uveitis are similar to those of children with uveitis in other JIA categories [30]. Data from the JPsA CARRA cohort also showed that uveitis was more common in children with early disease onset and ANA-positivity, but less frequently affected by skin disease [25].

Further evidence of the similarities between JPsA and non-psoriatic JIA has been provided by Butbul Aviel and colleagues in two studies. In the first [65], using a case-control design, the authors found similar clinical phenotypes and long-term outcome between 53 children with JPsA and 53 children with non-psoriatic JIA, matched by sex, age, date of diagnosis, and pattern of articular onset, with the exception of a higher frequency of dactylitis in patients with oligoarticular-onset JPsA and a lower frequency of hip and knee involvement in patients with polyarticular-onset JPsA. However, the editorial accompanying the article highlighted some study limitations, especially the small sample size, the clinical heterogeneity of the disease categories, and the lack of detection of demographic differences due to age- and sex-matching [8].

In the second study, Butbul Aviel et al. [66] reported the retrospective analysis of the charts of 122 patients who met the Vancouver criteria (definite or probable) or the ILAR criteria for JPsA. Their results suggest that JPsA may comprise 4 distinct groups (namely, oligoarthritis persistent or extended, RF-negative polyarthritis, RF-positive polyarthritis, and ERA) that are similar to non-psoriatic JIA in terms of disease onset and course, uveitis occurrence, response to treatment, and outcomes. The authors concluded that the coexistence of psoriasis may have little impact on the outcome and response to treatment of children with JIA and, therefore, psoriasis may be considered as a simple extra-articular manifestation, like uveitis, rather than a feature requiring distinct classification grouping.

With regard to genetic data, the diverse results observed in HLA association studies in JpsA [31,36,37] underscore the clinical heterogeneity among patients with JPsA, as defined by the ILAR classification.

### 4.3. The New Provisional PRINTO Classification Criteria

As mentioned above, the consideration of the shortcomings of the ILAR classification has led the international research network PRINTO to start a multinational effort aimed to revise the nomenclature and classification of JIA [57]. The project is intended to be conducted through a multistep strategy, based on the combination of the following consensus and evidence-based procedures [11]: (1) a Web-based Delphi consensus survey among 13 expert pediatric rheumatologists aimed to propose a framework for the revision of current ILAR criteria; (2) a nominal group technique (NGT) consensus conference among the same experts dedicated to the discussion and proposal of the preliminary PRINTO classification criteria; (3) the validation of the new criteria by means of a large-scale data collection of patients with new-onset JIA (currently ongoing); and (4) the evaluation of the results of validation analysis and the formulation of the final set of classification criteria in a second NGT consensus conference. The results of steps (1) and (2) have been recently published [11]. Notably, owing to the lack of consensus among the experts, no provisional definition was devised for JPsA and it was decided to postpone the decision regarding the categorization of this condition to the final stages of the project, provided that a set of clustering descriptors of this disorder is identified.

### 4.4. Authors’ Perspective

Overall, the available data and the studies published so far have not allowed for the precise definition of the clinical phenotypes and course of JPsA and its relationships with the other categories of JIA and adult PsA. As a result, considerable uncertainty and controversy exists around the concept of JPsA and the correct classification of this clinical entity. To improve the knowledge of JPsA there is the need to combine clinical information with the collection and analysis of genetic, immunologic, imaging, and multiomic data obtained from sufficiently large samples of patients.

## 5. Conclusions

Nowadays, it is well established that children with JPsA, as defined by ILAR criteria, do not constitute a single homogeneous population. This condition has been found to encompass at least two different subsets, one possessing the features of early-onset, ANA-positive JIA and the other sharing the characteristics of spondyloarthritis and bearing similarities with adult PsA. Based on this notion, it is being debated whether it is worth maintaining the distinctive category of JPsA in the JIA classification, as in the current ILAR scheme, or whether the presence of psoriasis (or psoriatic features) should be placed only among the descriptors of the articular phenotype.

Important insights into the characteristics of JPsA will be provided by the multinational data collection that is being conducted by PRINTO. It is expected that, based on this information and the subsequent consensus procedures, a better definition of the clinical association between arthritis and psoriasis or psoriatic features will be achieved. The understanding of the biologic relationship between arthritis and psoriasis in childhood requires well-designed analyses that integrate genetic investigations, clinical information, imaging procedures, and multiple pathophysiologic data.

## Figures and Tables

**Table 1:** Worldwide frequency of the ILAR categories of JIA.

ILAR JIA Categories	Frequency ^1^
Systemic arthritis	4.2–33%
Oligoarthritis	10.8–56.7%
Rheumatoid-factor-positive polyarthritis	1.3–11.2%
Rheumatoid-factor-negative polyarthritis	12.7–31.5%
Enthesitis-related arthritis	5.4–29.8%
Psoriatic arthritis	1.3–7.1%
Undifferentiated arthritis	3.7–10.4%

^1^ The reported frequencies refer to the percentage of all juvenile idiopathic arthritis patients. Data are extracted from Ref. [5]. Abbreviations: ILAR, International League of Associations for Rheumatology; JIA, juvenile idiopathic arthritis.

**Table 2:** Vancouver and ILAR classification criteria for JPsA (adapted from ref. [4,8]).

	Vancouver Criteria for JPsA ^1^	ILAR Classification Criteria for JPsA ^1^
Inclusion criteria	Definite JPsA: Arthritis plus typical psoriasis or Arthritis plus at least three of the following minor criteria: Dactylitis Nail pitting or onycholysis Psoriasis-like rash Family history of psoriasis Probable JPsA: Arthritis plus two of the minor criteria listed above	Arthritis plus psoriasis or Arthritis plus at least two of: Dactylitis Nail pitting or onycholysis Psoriasis in a first-degree relative
Exclusion criteria	None	Arthritis in an HLA-B27-positive male with arthritis onset after six years of age Ankylosing spondylitis, enthesitis-related arthritis, sacroiliitis with inflammatory bowel disease, Reiter syndrome, or acute anterior uveitis, or a history of one of these disorders in a first-degree relative Presence of IgM rheumatoid factor on at least two occasions at least three months apart Presence of systemic JIA

^1^ For both criteria, arthritis must begin before the 16th birthday, persist for at least 6 weeks, and be of unknown origin. Abbreviations: JPsA, juvenile psoriatic arthritis; ILAR, International League of Associations for Rheumatology; HLA-B27, human leukocyte antigen-B27; IgM, immunoglobulin M; JIA, juvenile idiopathic arthritis.

**Table 3:** Demographic and clinical features of patients with JPsA enrolled in the EPOCA study [5].

	Northern Europe	Western Europe	Southern Europe	Eastern Europe	North America	Latin America	Africa and Middle East	Southeast Asia
Epidemiologic data								
Frequency ^1^	35 (4.1)	40 (4.8)	88 (3.7)	54 (2.6)	37 (7.1)	13 (1.5)	37 (3.1)	5 (1.3)
Girls	25 (71.4)	27 (67.5)	63 (71.6)	36 (66.7)	24 (64.9)	8 (61.5)	17 (45.9)	2 (40)
Boys	10 (28.6)	13 (32.5)	25 (28.4)	18 (33.3)	13 (35.1)	5 (38.5)	20 (54.1)	3 (60)
Median (IQR) age of onset (years)	10.5 (6.2–12.9)	9.6 (3.9–11.4)	4.4 (2–10.4)	10.7 (6.4–13.6)	7.8 (5.6–11.9)	9.2 (5.9–11.7)	8.4 (4.5–11.8)	5.5 (2.3–12.)
Frequency of extra-articular features								
Psoriasis	27 (77.1)	24 (60.0)	52 (59.1)	37 (68.5)	29 (78.4)	8 (61.5)	24 (64.9)	5 (100.0)
Dactylitis	7 (20.0)	9 (22.5)	34 (38.6)	19 (35.2)	4 (10.8)	4 (30.8)	14 (37.8)	3 (60.0)
Nail pitting or onycholysis	3 (8.6)	8 (20.0)	18 (20.5)	13 (24.1)	7 (18.9)	5 (38.5)	12 (32.4)	0 (0.0)
Psoriasis in a first-degree relative	13 (37.1)	17 (42.5)	39 (44.3)	20 (37.0)	8 (21.6)	5 (38.5)	14 (37.8)	0 (0.0)
Clinical and laboratory features at visit								
Median (IQR) number of active joints	0 (0–1)	0 (0–1)	0 (0–1)	2 (0–5)	0 (0–1)	1 (0–4.5)	0 (0–2.5)	0 (0–4.5)
Enthesitis	6 (17.1)	5 (12.8)	3 (3.4)	5 (9.6)	2 (5.7)	1 (7.7)	8 (21.6)	0 (0.0)
Sacroiliitis	4 (11.4)	0 (0.0)	1 (1.1)	8 (14.8)	0 (0.0)	0 (0.0)	4 (10.8)	0 (0.0)
Median (IQR) ESR (mm/h) ^2^	7 (5–13)	10 (4.5–14)	10.5 (6–18)	9 (5–19)	7 (4–14)	17 (8.75–20)	12.5 (7.5–20.5)	20 (8–34)
Extra-articular complications								
History of uveitis ^3^	2 (7.4)	7 (21.9)	17 (25)	1 (2.6)	3 (10)	0	0	0

Data are extracted from the EPOCA study [5] and include unpublished data. Data are n (%) unless otherwise indicated. ^1^ Frequency refers to the percentage of cases of JPsA among all JIA patients enrolled in the EPOCA study in each geographic area [5]. ^2^ Erythrocyte sedimentation rate was available for 219 patients. ^3^ The frequency of uveitis was calculated in patients with at least 2 years of disease duration (*N* = 231) [5]. IQR, interquartile range; ESR, erythrocyte sedimentation rate.

## Data Availability

Not applicable.
